# Itching

**Published:** 1899-11-18

**Authors:** 


### ITCHING.

Of all things itching is the worst to put up with, and it is truly said by Mr. Willmott Evans[1] that there is no symptom connected with diseases of the skin which causes more trouble and discomfort than this does. Compared with it pain is a small matter, and patients suffering itching will scarify themselves with their nails, drawing blood at every stroke, in their efforts to obtain some degree of diminution of the irritation, which utterly incapacitates them for work bodily or mental. Itching, however, is but a symptom, and whether the cause of the pruritus lie in the skin itself, or in some internal disorder, such as diabetes, jaundice, or Bright's disease, the first indication is to treat the cause. In addition, however, to the mere treatment of the exciting cause, great attention should be paid to the general health, and any article of food must be avoided which the patient finds to be associated with aggravation of the irritation. The bowels must be kept open, and, on the whole, salines seem the best aperients for this purpose. Then, of course, the disease, whether of skin or of other parts on which the itching depends, must be treated; but, putting this on one side, it is as well to consider what can be done for the itching itself, considered apart from its probable causation. It is, first of all, essential that no external irritation from clothes or soap should be permitted. Many people cannot wear wool in any form next the skin, and in many cases no small measure of relief will result from the wearing of pure linen in contact with it, even though, for the sake of warmth, woollen garments may be worn outside. Among curative agents baths take the first place, the most efficient being those which contain alkalies, a quarter to half a pound of carbonate or bicarbonate of sodium or potassium being used in the ordinary 30-gallon bath. A borax bath, or one of potassa sulpharata, may be used, four ounces of either of these salts being added to the 30 gallons of water. Starch or bran baths may be used, separately or combined with the alkaline bath; and in severe cases a continuous bath may be employed with much benefit, for several days or even weeks. Again, Turkish baths are sometimes useful, perhaps from the free sweating which they induce, in which respect they have a much more than merely local action. Of all lotions those containing carbolic acid are the most useful, and the best strength is one of the acid to 60 of water, or alkalies or borax may be added. Dilute acetic acid with an equal quantity of water is sometimes useful, as also is lead lotion with glycerine, chloroform lotion (a drachm to a pint), or the various forms of tar lotion, although the staining of the skin is an objection to the employment of the latter. If the surface affected be small ointments are sometimes more convenient, and these may contain carbolic acid, grains 5 to 30, with zinc ointment; ichthyol, grains 30 to 180, with calamine; resorcin grains 15 to 60, with oxide of zinc; or beta-naphthol, grains 20; all the above quantities being made up to an ounce with paraffin ointment. Mercury may be employed over small surfaces in the form of ammoniated mercury with oleate of zinc and paraffin ointment, or of the dilute nitrate of mercury ointment diluted with paraffin ointment. A cut lemon rubbed on the part sometimes relieves, and in pruritus ani a cocaine or a cocaine and morphine suppository often has a striking result.

[1] Treatment, Oct. 26.

